# Seeking consensus on dilemmas related to euthanasia in dementia based on an advance directive: a Delphi study from a medical, ethical and legal perspective

**DOI:** 10.1136/jme-2024-110276

**Published:** 2025-01-28

**Authors:** Marike E de Boer, Djura O Coers, Eefje M Sizoo, Danique M J ten Bokkel Huinink, Carlo J W Leget, Cees M P M Hertogh, DALT project group, Nettie Blankenstein

**Affiliations:** 1 Medicine for Older People, AmsterdamUMC Location VUmc, Amsterdam, The Netherlands; 2 Aging & Later Life, Amsterdam Public Health Research Institute, Amsterdam, The Netherlands; 3 Care Ethics, University of Humanistic Studies, Utrecht, The Netherlands

**Keywords:** Euthanasia, Dementia, Decision Making, Ethics- Medical, Advance Directives

## Abstract

Euthanasia in dementia based on advance euthanasia directives (AEDs) is possible within the Dutch Euthanasia law. Yet, physicians struggle with the responsibility of interpreting the law’s open norms in cases of advanced dementia, which includes the fulfilment of the due care criteria. This Delphi study aims to analyse arguments and seek consensus from medical, ethical and legal perspectives on ethical dilemmas in such cases. Thirty participants, equally divided in expertise, took part in a three-round Delphi with a total of 11 statements on ethical dilemmas. Despite differences in opinions and argumentations between panellists, consensus was reached on seven statements regarding different topics. Consensus was reached that the (behavioural) expressions of a person with dementia should be considered throughout the progression of decision-making disabilities. In such cases, a wish to live should be prioritised over an AED. Although substitute decision-making is not an option in case of euthanasia requests, both people around the person with dementia as well as their AED can be supportive in the decision-making process. Advance directives with formulations such as ‘if I have to admitted to a nursing home, then I want euthanasia’ are found to be infeasible. At all times, it is important to pay attention to alternatives to euthanasia, which includes following existing guidelines on problem behaviour. Physicians may benefit from the arguments pertaining to dilemmas encountered and the fulfilment of the due care criteria to either justify their decisions in euthanasia cases based on an AED, or to support decisions to refrain from euthanasia.

## Introduction

 Since 2002, the Netherlands has regulated euthanasia and physician-assisted suicide (in this article referred to as ‘euthanasia’) under the ‘Termination of Life on Request and Assisted Suicide (Review Procedures) Act’ (commonly known as Euthanasia law).^1^ The Euthanasia law defines the conditions under which physicians are permitted to conduct euthanasia without legal consequences, provided they report the case to the Regional Euthanasia Review Committees (RERC).^2^ The cornerstone of the conditions within the Euthanasia law is formed by the ‘due care criteria’, which must be adhered to (see box 1).

Box 1 **The due care criteria for euthanasia or assisted suicide as stated in the Euthanasia law**

**The physician must:**

- Be satisfied that the patient’s request is voluntary and well considered.
- Be satisfied that the patient’s suffering is unbearable, with no prospect of improvement.
- Have informed the patient about their situation and prognosis.
- Have come to the conclusion, together with the patient, that there is no reasonable alternative in the patient’s situation.
- Have consulted at least one other, independent physician, who must see the patient and give a written opinion on whether the due care criteria set out above have been fulfilled.
- Have exercised due medical care and attention in terminating the patient’s life or assisting in the patient’s suicide.

The Euthanasia law also allows an oral request (criterion 1) to be replaced by a written advance euthanasia directive (AED) (article 2.2 Euthanasia law). In such cases, the due care criteria apply ‘mutatis mutandis’ (roughly translated as ‘not literally, but without misunderstanding’).^3^ This article opened doors for people with decision-making disabilities, such as advanced dementia, to receive euthanasia. However, this possibility ignited significant controversy^4^,^6^ and a broad legal, ethical and practical debate.

In practice, physicians bear the responsibility for interpreting the open norms of the euthanasia law^7^, including the fulfilment of the due care criteria. However, the rooting of the due care criteria lies in end-of-life assistance of decisional capable patients which makes physicians struggle with assessing the due care criteria for euthanasia based on aeds in the context of patients with advanced dementia.^8 9^ In this context, the number of AED-based euthanasia cases performed is low (6 in 2022).^10^

Physicians face multiple ethical dilemmas in the actual practice of dealing with AEDs in dementia care, dominantly related to the increasing challenges in communicating with the patient as the disease progresses. Hampered communication makes validation of ‘anticipatory choices’ challenging, in terms of determining the exact moment for the AED’s effectiveness and the potential for changed preferences.^11^,^14^ It becomes challenging to balance the wishes laid down in an AED on the one hand and giving weight to ((non-) verbal) expressions and behaviour of the person with advanced dementia on the other hand.^1112 14^,^17^ At the same time, determining unbearable suffering and discussing alternative treatment options become highly complex without meaningful patient communication.^8 9 12 13 18 19^

Despite provided guidance from the Royal Dutch Medical Association (KNMG),^20^ Reports from the rerc on specific cases,^12 21^ As well as a supreme court’S ruling, on a case of aed-based euthanasia,^22 23^ Physicians continue to face complexities in applying the open norms of the euthanasia law in actual practice. In the light of these challenges and with the overall aim to provide additional guidance, we conducted a delphi study to examine position statements regarding ethical dilemmas associated with euthanasia requests of Patients With dementia and an aed. This study aims to analyse arguments and seek consensus from medical, ethical and legal perspectives.

## Methods

This Delphi study is part of a comprehensive research project, titled: *Euthanasia in patients with dementia and an advance euthanasia directive: towards practical guidance*. The overall objective of the DALT project is to develop a research-based practice guidance for physicians, offering approaches to handling AEDs in patients with advanced dementia.

### Design

This study used a three-round modified Delphi consensus approach^24^,^26^ to gain insight and build consensus. The method aims to determine the extent of agreement about the statements by seeking opinions from individuals knowledgeable on the subject through a series of structured questionnaire rounds with controlled feedback.^27 28^ Each questionnaire included pseudomised results from the previous round(s), and participants were asked to consider these results when providing their replies in subsequent rounds.

### Panellists

The DALT (Dementia, Advance directives, and Life Termination) project group compiled a list of more than 60 potential Delphi panellists, selecting individuals with expertise in medical, legal or ethical fields and a strong understanding of the topic of AED-based euthanasia in dementia. This diverse composition, based on knowledge and supplemented with an internet search, aimed to capture a broad perspective and promote consensus generalisation. The list included potential legal and ethical panellists from Belgium, due to the country’s euthanasia legislation and their proficiency in Dutch. To uphold independence and avoid conflicts of interest, experts affiliated with stakeholder organisations or advocacy groups were excluded from participation. Inclusion was limited to panellists mastering the Dutch language.

Purposive sampling, ensuring diversity in expertise and geographical distribution, was conducted via email until a plausible number of 31 panellist was included. In case one of the initially approached panellists declined participation, the next potential panellist with a similar profile and who met the criteria was approached. Two panellists dropped out, after initial agreement, for all Delphi rounds due to time constraints or other reasons for unavailability. Prior to the start of the study, all panellists received written information detailing the Delphi process, procedure and timing. Consent was obtained from all at the start of the first round. Pseudonymity of panellists was strived for throughout the study, with names disclosed only in the acknowledgement section of this article if desired.

### Consensus criteria

We assessed agreement using a 4-point Likert scale (agree, agree more than disagree, disagree more than agree and disagree), with an option for ‘no expertise’. Panellists were asked to provide arguments for their chosen agreement level. Motivated by the topic’s sensitivity, consensus was predetermined as at least 80% agreement on the scale and aligned argumentation.

### Development of statements and Delphi rounds

Three Delphi rounds were conducted between May and July 2023. Questionnaires were administered using Castor Electronic Data Capture Software.^29^ Each panellist received a secure link to the questionnaire via email, ensuring pseudomised completion. For the first round, 10 statements were drafted by the DALT project group focusing on dilemmas related to AED-based euthanasia in dementia. These statements were formulated based on existing literature, including results from other parts of the DALT project,^2 4 9 14 20 22 23 30 31^ piloted and refined through discussions within the DALT project group. Explanatory text boxes were included in the questionnaire to support the context of the statements, without these boxes being evaluated. Statements could be modified or added after each round.

In each round, panellists indicated their level of agreement with each statement and provided supportive argumentations. In subsequent rounds (two and three), both the level of agreement and supportive argumentations were returned anonymously and unedited (excluding privacy-sensitive information), enabling panellists to review and consider their input. Statements were omitted from scoring in the next round once consensus was reached and argumentations remained stable over consecutive rounds. Member checking was employed by including statements achieving consensus to the next round without rescoring, but with an option to add arguments. If the number of responses indicating a lack of expertise exceeded five, statements involved were rescored. The Delphi process was concluded after three rounds.

### Data analysis

Analysis incorporated both qualitative thematic analysis and quantitative techniques. Consensus percentages were calculated for both the overall panellist group and separately for the medical, ethical and legal panellists. Responses to statements scored on the 4-point Likert scale were grouped into two categories for analysis: disagreement (‘disagree’ and ‘disagree more than agree’) and agreement (‘agree more than disagree’ and ‘agree’); the ‘no expertise’ responses were excluded from the calculation. Consensus was assessed by comparing the calculated percentages of agreement or disagreement against a predefined consensus threshold of 80% based on sensitivity of the subject. Thematic analysis was applied to arguments per statement, supported by the software tool MaxQDA.^32^ Responses were coded independently by three researchers (MEdB, DOC and EMS) to ensure analysis reliability and validity, resolving discrepancies in pairs (MEdB and DOC; MEdB and EMS) through discussion. Arguments were categorised into those supporting the statement and counterarguments. If subdivision was not possible, overall themes were presented for comprehensive insight. A final draft of the outcomes was reviewed and discussed within the project group.

## Results

**Figure 1:**
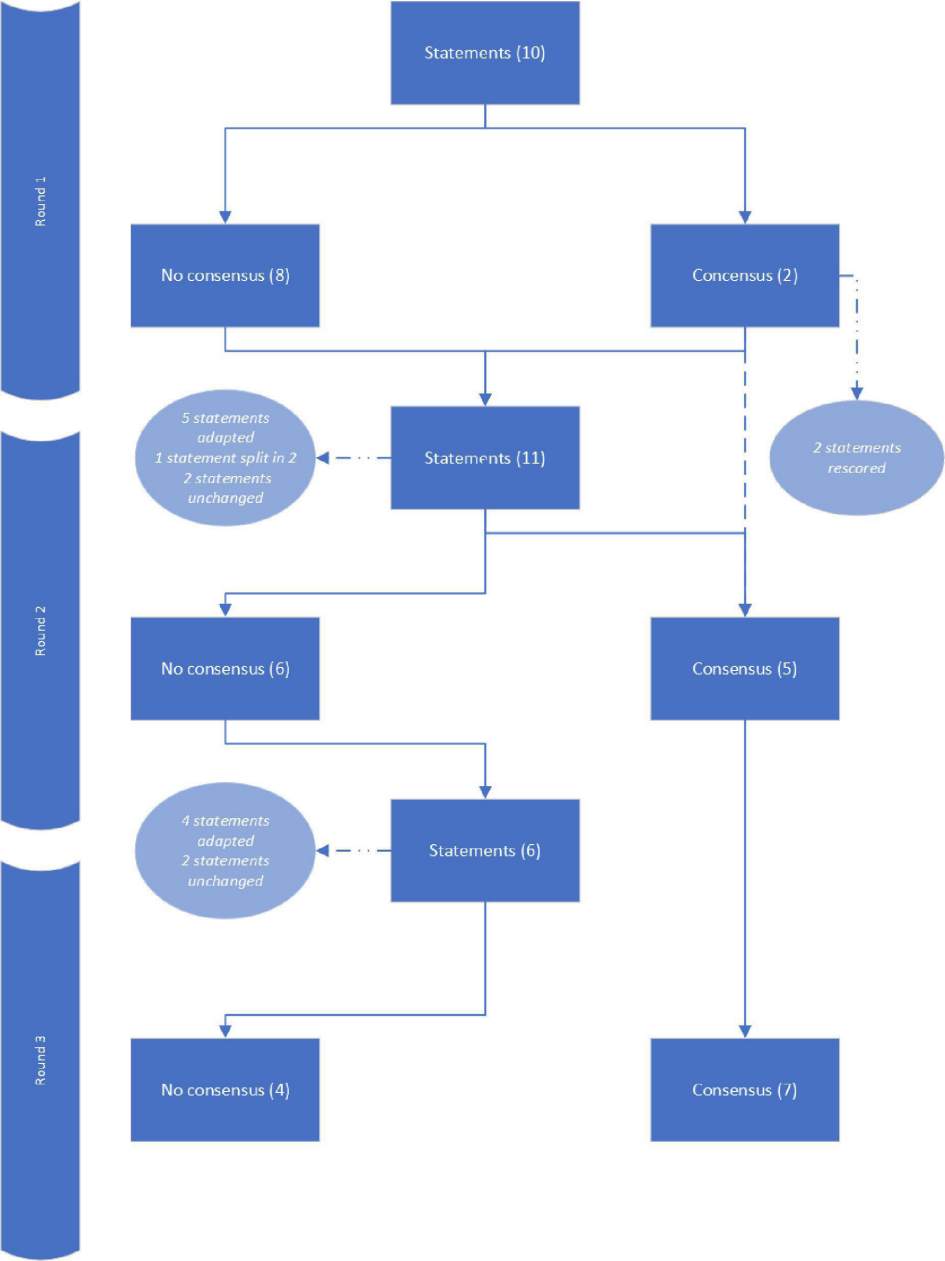
Flowchart statement consensus over Delphi rounds*. * For all statements with consensus both a summary and full arguments were provided in the next round. In case arguments pointed in the same direction as those of the previous round, statements were not re-scored in the next round.

Twenty-six of 30 panellists (87%) participated in all three rounds of this delphi study and per round 87%–97% Of panellists responded (see online supplemental appendix For details on participation per round and panellists’ Characteristics). The study started with 10 and ended with a total of 11 statements that were proposed and scored. Seven statements reached consensus after Three Rounds (see figure 1).

Consensus results of final Delphi rounds are presented for all statements, along with arguments of all rounds in terms of supporting and counterarguments (unless otherwise specified). Additional data on changes in statements and consensus over rounds can be found in the online supplemental appendix. Statements were categorised under ‘applicability of article 2.2’, ‘communication’, ‘decision-making support’ and ‘problem behaviour’.

### Applicability of article 2.2

Statement 1 (table 1) on the interpretation ‘mutatis mutandis’ of article 2.2 of the Euthanasia law did not reach consensus, except among medical panellists. The main difficulty for physicians arises from the lack of communication with individuals with advanced dementia, making it challenging, if not impossible, to fulfil the due criteria.

**Table 1:** Application of article 2.2 Euthanasia Law—Statement, consensus percentages and arguments

Statement*	(Sub)group (n)	Consensus (% Agree; round)†
1. The wording that, in the case of the application of article 2.2 of the euthanasia law, the due care criteria apply ‘Mutatis mutandis’, Does not provide sufficient clarity for physicians in practice	Overall (26):	73%; 3
	Legal panellists (9)	56%
	Ethical panellists (8)	75%
	Medical panellists (9)	89%
Themes‡§: 1. Clarity of the phrase ‘Applicable mutatis mutandis’ Cannot be provided in the case of advanced dementia. Quote → “the possibility of dialogue disappears, making it much more difficult to fulfill the first four due care criteria. Physicians struggle (too much) with the interpretation of the law in these types of situations (a lot of uncertainty and moral stress) and require more support” (M) 2. The interpretation/meaning of the phrase ‘Applicable mutatis mutandis’ Can be sufficiently derived from other documents Quote → “from the parliamentary history, the ‘Case-law’ Of the rtes, and the supreme court ruling of 2020, it appears that the meaning of this phrase is: The criteria are applicable to the extent that they can be applied in the case of a Patient With advanced dementia.”(E) 3. Interpretation of the open norms in the law is up to the (medical) field Quote → “…all due care criteria in the euthanasia law are formulated as open norms to which the evolving practice of consultation- and assessment should provide further interpretation”(E) Quote → “Mutatis mutandis is interpretation, and this will differ among doctors.” (E)		

* Consensus on statements highlighted in light grey; no consensus on statements highlighted in dark grey.

† Round numbers represent the number of completed rounds with a maximum of 3.

‡ Due to varying interpretations of this statement among panellists, the presentation of these data focuses on relevant themes rather than including supporting and counter arguments.

§ The capitals after the quotes refer to the sources in terms of legal (L), ethical (E) or medical (M) panellist.

Some panellists suggest that interpretation of ‘mutatis mutandis’ should be left to medical expertise rather than specified in the law, while others propose deriving it from documents like the RERC code of practice and existing case law.

### Communication

All four statements in table 2 address communication issues in AED-based euthanasia cases. While article 2.2 permits replacing an oral request with an AED, the overall opinion (table 2, statement 2, consensus) is that the current expressions and behaviours should never be ignored. This is seen as “cross-border*”* (M) and “not good practice of care” (M). Legal experts argue against withdrawing euthanasia requests when decisional capacity is lost. However, other panellists argue that current expressions of the person with dementia are vital in determining suffering, though interpretation can be difficult.

**Table 2:** Communication—Statements, consensus percentages and arguments

Statement*	(Sub)group (n)	Consensus (% Agree; round)†
2. The current expressions and Behaviours Of the (decisional incapacitated) Patient Should never be ignored	Overall (25):	87%; 2
	Legal panellists (7)	71%
	Ethical panellists (9)	89%
	Medical panellists (9)	100%
Supporting arguments‡§: Ignoring current expressions and Behaviours Is not ‘Good practice of care’ Ignoring current expressions and Behaviours Does not do justice to the current person Current expressions and Behaviours Can reflect the actual wish Quote → “Ignoring is a violation of who they are now as a person”(E) Quote → “Ignoring these expressions is cross-border to me”(M) Counterarguments¶§: A decision for euthanasia made by a competent individual cannot be revoked if they later become incompetent Quote → “if that [Revoking a euthanasia request] Were possible, it would undermine the meaning of article 2.2 of the euthanasia law (wherein the written declaration replaces the current request)”(l) Overarching arguments: Current expressions and Behaviours Must be interpreted and valued (frequency, context, intensity, consistency, etc, Are factors to consider) Current expressions and Behaviours Are important in determining (unbearable) suffering Active life termination in cases of physical resistance is ‘Morally very problematic’ And burdensome for the physician Quote → “The complexity lies in the interpretation of expressions and behaviors!”(E) Quote → “Ignoring is never allowed, interpreting is another thing” (E)		
3. A wish to live (deduced from current expressions or Behaviours—However inarticulate) must take precedence over a written euthanasia declaration	Overall (25):	82%; 2
	Legal panellists (7)	57%
	Ethical panellists (9)	89%
	Medical panellists (9)	100%
Supporting arguments‡§: A wish to live must be respected, also from a human rights perspective and the ‘Protection’ Of people with dementia If, based on the wish to live, the physician is not convinced of the request or of the unbearable and hopeless suffering, euthanasia cannot be performed A wish to live should, also considering its irreversibility, be regarded as a contraindication for the implementation of euthanasia From the perspective of the physician: “Can one expect them to end your life when you express a desire to live?” Quote → “the current person deserves ‘Protection’ Compared to their former self… Who… In the past may not have been able to fully anticipate who they would be with dementia."(E) Quote → “When in doubt, don't… Only 100% Certainty of a current wish for death is good enough for me”(M) Counterarguments¶§: When the wish to live prevails, the euthanasia directive would be an ‘Empty shell’ People do not always agree with a Prioritisation Of their current wish for life in their advance directive Quote → “can an unarticulated wish replace a voluntary and well-considered request discussed with others? […] [This replacement] Threatens to make article 2.2 euthanasia law meaningless”(L) Quote → “if there is inconsistent behaviour/expressions, in my opinion, it is Unbalanced To automatically take the expressions indicating a desire to live more seriously than the wish for death”(L)		
4. Current (non-verbal) communication between doctor and patient is a prerequisite for proceeding with euthanasia	Overall (25):	59%; 3
	Legal panellists (8)	25%
	Ethical panellists (8)	63%
	Medical panellists (9)	89%
Supporting arguments‡§: Essential for determining the current autonomous wish of the Patient Without some form of communication the unbearableness of suffering cannot be determined Boundaries can be shifted, which can also postpone the wish for death The Patient Must retain the right to refrain from euthanasia, even through resistance Physicians should not be expected to end the life of someone who does not understand what is happening Quote → “there must be some form of communication between the physician and the Patient, Which cannot be misunderstood, from which the physician can deduce that the situation is unbearable for the Patient At this moment, and that the current situation is the one for which the Patient Drafted the advance directive.” (M) Quote → “There must be some echo of the wish to die and of the experience of unbearable suffering."(E) Counterarguments¶§: Article 2.2 of the euthanasia law is precisely intended for situations where communication is no longer possible The advance directive includes the fundamental/critical interests (priorities) and these take precedence Quote → “Requiring this…, implies that article 2.2 of the euthanasia law would be a meaningless provision”(L) Quote → “the Patient Himself has expressed his own his opinion about the prioritization [Of interests] In his advance directive], and it is not appropriate for a physician to IMpose their own ethics or those of the majority of their profession onto the Patient” (E)		
5. If mutual (non-verbal) communication is not possible, the physician can obtain clarity through a broad orientation with consultation from multiple sources (eG, Family, caregivers And Other healthcare professionals) regarding whether there is agreement between the patient’S current expressions and behaviours and their earlier advance directive	Overall (25):	64%; 3
	Legal panellists (8)	88%
	Ethical panellists (8)	50%
	Medical panellists (9)	56%
Supporting arguments‡§: Consultation with multiple sources is helpful in obtaining clarity about: The Patient’S wish, the scope and applicability of the advance directive And the suffering As an addition to (previous) communication with the Patient Themselves, which should be central It is a resource, but the physician remains ultimately Quote → “Broad consultation with multiple sources is desirable to make a well-informed decision."(L) Quote → “if consensual assessment of the suffering takes place with people with a different background and relationship to the Patient And ultimately a conclusion is drawn that is unambiguous, then in my opinion this is a good method”(M) Counterarguments¶§: The interpretation of suffering and therefore the wish for death is subjective; broad orientation with consultation from multiple sources cannot replace communication with the Patient About the wish for death Quote → “This is a slippery slope. People start filling in for others.” (M); “The slippery slope comes when the suffering is less clear or not continuously present. Or when not all people involved agree”(M)		

* Consensus on statements highlighted in light grey; no consensus on statements highlighted in dark grey.

† Round numbers represent the number of completed rounds with a maximum of 3.

‡ This concerns respondents who scored ‘Agree’ or ‘More agree than disagree’ with the statement.

§ The capitals after the quotes refer to the sources in terms of legal (L), ethical (E) or medical (M) panellist.

¶ This concerns respondents who scored ‘More disagree than agree’ or ‘Disagree’ with the statement.

In case current expressions and/or behaviours are interpreted as a ‘wish to live’, there is overall consensus (table 2, statement 3) that this wish should prevail over the AED although some panellists feel that this would undermine the intentions of article 2.2, and that some people writing an AED might disagree with this kind of prioritisation of their actual ‘wish to live’. The prevailing sentiment is that *“*the current person deserves ‘protection’ against his former self” …who… “in the past may not have been able to fully foresee who they would be with dementia” (E).

Opinions diverge on whether actual patient–physician communication should be a precondition for considering euthanasia (table 2, statement 4, no consensus). Advocates argue that communication is essential: *“*People underestimate how desperate you must be to want to die. Only an advance directive cannot convey to a doctor that this is truly the moment, that the suffering is now too severe, that the despair is too great” (M). Alternatively, the counterargument is that article 2.2 is created for situations in which patient communication is no longer possible, with the most far-reaching argument being that the AED should prevail over patient’s actual expressions. These differences in opinion are referred to as “personal ethics*”* (E) with fundamental different opinions on “the authority of the advance directive for dealing with the individual in the advanced stage of dementia” (E).

The diverse personal ethics are evident in varying opinions (table 2, statement 5, no consensus) regarding the role of family and other (informal) caregivers in determining the alignment of behaviour and expressions of the person with dementia with their AED. While some believe that consulting others contributes to the interpretation of expressions in relation to a person’s wishes and suffering, others emphasise the essence of the person’s ‘voice’, especially when it comes to interpreting their situation as one of (unbearable) suffering.

### Decision-making support

Table 3 displays all statements related to support in decision-making, in addition to patient communication, which includes the role of decisional (in)capacity of the person with dementia, the role of the AED and the role of representatives.

**Table 3:** Support in decision-Making—Statements, consensus percentages and arguments

Statement*	(Sub)group (n)	Consensus (% Agree; round)†
The role decisional (in)capacity		
6A. In general, strict requirements for decisional capacity apply to oral requests for euthanasia, where in the case of dementia, it makes no difference whether the Patient In question has a written Advance euthanasia directive Or not.‡	Overall (25):	25%; 3 (76% Disagree)
	Legal panellists (9)	0%
	Ethical panellists (7)	43%
	Medical panellists (9)	33%
Themes‡§: 1. Strict requirement decisional capacity in case of an oral request: A strict/stringent requirement/ high threshold Quote → “given the complexity of such a decision, the level of care must be high, and in my opinion, this is achieved with a high threshold for mental capacity”(M) A requirement that is too strict is in conflict with human rights. Quote → “that is in conflict with the convention on the rights of persons with disabilities, under which the wishes of people with intellectual disabilities must be taken seriously and it should not be hastily concluded that the person lacks [Decisional] Capacity. I personally fully agree with that.”(L) 2. The role of an aed in determining decisional capacity in case of an oral request: An aed does not affect (actual) decisional capacity Quote → “for an oral request, high standards of decisional capacity apply regardless of whether one, two, or multiple prior aeds have been made.”(L) The aed does not play a role in the assessment (situation of decisional capacity) Quote → “An aed is not necessary in the event that the Patient Can still make a (valid) oral request” (L) The aed can replace the oral request (situation of decisional incapacity) Quote → “the aed is also important in the event that a Patient With dementia makes an oral request. Under certain conditions, the aed can even replace the oral request.”(L) The aed can support the assessment (situation of uncertainty about decisional capacity) Quote → “The aed can complement a certain amount of reduced decisional capacity”(E) Quote → “the existence of an aed makes the conversation about the request for euthanasia easier to interpret. Consistence of the wish and the story of the Patient Can help in the assessment of decisional capacity in this matter”(M)		
The role of an aed		
6b. If a Patient With dementia makes an oral request for euthanasia, a written Advance euthanasia directive Can be supportive in the discussion and decision-making regarding that request.	Overall (25):	92%; 2
	Legal panellists (8)	88%
	Ethical panellists (8)	100%
	Medical panellists (9)	89%
Supporting arguments¶§: Support by an aed is self-evident, provided that the aed has been composed voluntarily and well-considered The person with dementia has to be given a voice; and possible changes in their wishes have to be taken into account The aed: Helps to start the conversation Strengthens and provides insight into the oral request Underlines the ‘Well-considerateness’ Of the oral request Quote →“A previous aed can assist in the assessment of the ‘Well-considerateness’ Of the actual request”(M) Quote →“if the Patient Is capable of making an oral request, that request is leading, and the living will plays a minor role”(J) Quote → “If the Patient Has previously considered euthanasia and put it into writing, it provides additional insight into the Patient’S will/wishes”(J) Counterarguments**§: In advanced dementia the aed is leading, with a supportive role for the oral request Quote → “If an aed is considered only as supportive, the rationale for its drafting is invalidated”(E)		
7. Advance directives with formulations such as ‘If i have to be admitted to a nursing home, then i want euthanasia’ Are infeasible	Overall (26):	92%; 2
	Legal panellists (8)	88%
	Ethical panellists (9)	89%
	Medical panellists (9)	100%
Supporting arguments¶§: Further substantiation of this formulation is essential There is possibly a distorted perception of nursing home care Unbearable suffering is required which is not reflected in such an aed People can adjust their wishes; you only know if it’S unbearable when you’Re in the situation The wording places too big a demand on caregivers. Quote → “…I also find it to be an impossible demand on caregivers: They sometimes push themselves to the limit and beyond because they don’T want to sign the death sentence of their loved one (because that’S what you’Re Essentially doing when you indicate that you can no longer provide care at home)”(m) Quote → “Suffering should not be determined by the place of residence, but by one’S own physical and mental condition”(M) Quote → “Such a formulation is far too coarse-grained and goes back to prejudices related to the bad reputation of nursing homes”(E) Counterarguments**§: An aed, even with such a formulation, needs to be interpreted; further substantiation of this wording can be determined through alternative means. The person should be able to refuse admission to a nursing home Quote →“is a nursing home a reasonable alternative that impedes euthanasia performance? … This admission could after all prolong the suffering the Patient Tries to avoid (l)”		
The role of representatives		
8. Substitute decision-making is not an option for highly personal decisions such as euthanasia or assisted suicide (In substitute decision-making, the decision is taken over by the legal representative)	Overall (26):	88%; 2
	Legal panellists (8)	88%
	Ethical panellists (9)	89%
	Medical panellists (9)	89%
Supporting arguments¶§: The decision to end one’S life is so profound and irreversible that it can only be made by the individual themselves In the case of substitute decision-making, changes in the client’S wishes cannot adequately be taken into account. The underlying motivation of a representative is difficult to determine Substitute decision-making is legally not possible when it comes to euthanasia or assisted suicide. Quote → “It’S really about one’S own will, not that of others.”(L) Counterarguments**§: Substitute decision-making is (only) an option when explicitly documented in a written advance directive Substitute decision-making is in general no option, but should not be ruled out categorically; it should be preserved for extreme cases Quote → “…In some extreme cases, there may ultimately be no alternative. However, clinging to that principle in such instances ultimately reduces us to executioners, surpassing the bounds of humanity.”(L) Overarching arguments§: Representatives can support the physician, and vice-versa, the physician can also consult the representative Quote → “The physician may consider the opinions of the Patient’S loved ones in the deliberations, but these can never replace the requirement of a voluntary and well-considered request from the Patient” (M,l) Quote → “in the event of decisional incapacity, the representative can play a supportive role in interpreting the advance directive and the unbearable and hopeless nature of the suffering (e)” Quote → “a representative cannot make such a decision on behalf of a Patient. However, a representative can assist in clarifying previous or current expressions of the Patient.”(L)		

* Consensus on statements highlighted in light grey; no consensus on statements highlighted in dark grey.

† Round numbers represent the number of completed rounds with a maximum of 3.

‡ Due to the complexity of this statement (two aspects within the statement + different interpretations depending which situation of decisional capacity was in the mind of the respondent), presentation of data focuses on relevant themes rather than supporting and counter arguments.

§ The capitals after the quotes refer to the sources in terms of legal (L), ethical (E) or medical (M) panellist.

¶ This concerns respondents who scored ‘Agree’ or ‘More agree than disagree’ with the statement.

** This concerns respondents who scored ‘More disagree than agree’ or ‘Disagree’ with the statement.

AED, advance euthanasia directive.

#### The role of decisional (in)capacity of the person with dementia

Decisional (in)capacity is a relevant but complex issue in the context of AED-based euthanasia (table 3, statement 6a, no consensus). In our study, panellists argue that the requirements for decisional capacity should be strict due to the complex and irreversible nature of euthanasia. However, counterarguments suggest that overly strict requirements may potentially conflict with human rights, as the wishes of people with disabilities should always be taken seriously regardless decisional (in)capacity. As explained by one of our medical panellists, decisional capacity is particularly relevant at the moment of the euthanasia request: “If there’s no statement [AED], the physician must ensure the person has capacity at the time of the request. If there is a statement, the oral request doesn’t necessarily need to be tested for decisional capacity, but rather on whether the situation indeed matches what was previously described in the statement.” (M).

#### The role of the advance directive

Consensus (table 3, statement 6b) among medical, ethical and legal panellists favours the supportive role of AEDs in the consultation and decision-making process regarding oral euthanasia requests from individuals with dementia. They are considered valuable in “giving a voice*”* (E) to those with advanced dementia. However, the complexity resides in determining: “which conclusions are drawn from this [advance directive]?” (L). AEDs stating ‘if I have to be admitted to a nursing home, then I want euthanasia’ are deemed infeasible (table 3, statement 7, consensus), with the primary argument being “You cannot automatically equate ‘admission to nursing home’ with ‘unbearable suffering’’ (E) (second due care requirement).

#### The role of representative(s)

While some panellists hesitate to categorically reject it, consensus (table 3, statement 8) is reached on ruling out substitute decision-making by representatives for highly personal decisions like euthanasia: ‘The decision to end a life is so profound that it can only be made by the individual themselves” (E). This does not imply that representatives have no role whatsoever. It is argued that representatives certainly can fulfil a supportive role in decision-making about euthanasia in dementia, by interpreting expressions of the person of dementia, their suffering, or meaning of their AED.

### Problem behaviour

Table 4 Includes statements on problem behaviour as a cause of suffering.

**Table 4:** Problem Behaviour—Statements, consensus percentages and arguments

Statement*	(Sub)group (n)	Consensus (% agree; round)†‡
Problem behaviour		
9. If suffering arises from problem behaviour, application of the professional guideline problem behaviour is strongly recommended to be able to assess whether or not there is hopelessness of suffering	Overall (21):	96%;3
	Legal panellists (5)	100%
	Ethical panellists (7)	100%
	Medical panellists (9)	89%
Themes§ 1. “That’S what it’S there for” Quote → “‘Applying the guideline for problem behavior means adopting a methodical approach to a complex case. That is part of carefulness”.(M) 2. First try to treat the suffering before taking the step towards euthanasia. Quote → “Euthanasia remains the ultimate remedy. Good care means trying to reduce problem behaviour”(L) Quote → “It fits with medical careful and professional acting to treat/fix ‘Treatable suffering’ Prior to moving on to euthanasia” (M) 3. Proportionality Quote → “carefully ruling out possible causes of problem behaviour should be done first, whether all pharmacological and non-pharmacological treatment option have been tried, and whether the problem behaviour is truly refractory”(M) Quote →“That seems logical in itself, as long as it’S not a way to avoid euthanasia issues”(L)		
10. In case of refractory problem behaviour (Ie, Problem behaviour that cannot be alleviated by optimal care and treatment), it is the professional standard to also consider and discuss the options outlined in the guide on palliative sedation in case of refractory problem behaviour	Overall (21):	84%;3
	Legal panellists (5)	80%
	Ethical panellists (7)	71%
	Medical panellists (9)	100%
Themes§ 1. Part of good medical practice. Quote → “as a physician, you must consult, consider, and discuss the relevant guidelines and guides to do what you are supposed to do as a good care provider” (L) Quote → “That’S part of your professional approach. Sedation is a treatment option that must be seriously considered”(M) Quote → “you must explore all reasonable possibilities to alleviate suffering. The guide on palliative sedation can offer a perspective to relieve unbearable suffering”(E) 2. Palliative sedation can be a reasonable alternative (but not always) Quote → “…Palliative sedation may offer possibilities to control the symptoms”(M) 3. Remarks/ethical issues Quote →“intermittent sedation is a good option for refractory problem behavior. It falls under normal medical practice (m) Quote → “[Continuous] Palliative sedation is considered acceptable only when the life expectancy is less than 14 Days; Otherwise, sedation would be a disguised form of life termination”(E)		

* Consensus on statement highlighted in green; no consensus on statements highlighted in orang*e*.

† Round numbers represent the number of completed rounds with a maximum of 3.

‡ Consensus is reached when respondents claiming to have no specific expertise on this guideline are left out of the analysis.

§ Due to varying interpretations of this statement among respondents the presentation of this data focuses relevant topics rather than including supporting and counter arguments.

§, The capitals after the quotes refer to the sources in terms of legal (L), ethical (E) or medical (M) panellist.The capitals after the quotes refer to the sources in terms of legal (L), ethical (E) or medical (M) panellist.

Some legal and ethical panellists reported their lack of expertise with regard to the content of both the ‘guideline problem behaviour’ and the ‘guide on palliative sedation’. Nonetheless, within the expertise group, consensus was reached on both statements about usefulness of these guidelines in cases of problem behaviour (table 4, statements 9 and 10).

Medical panellists stated: “Suffering and problem behaviour are connected to each other” (M)*,* whereby the “guideline helps to make a proper analysis and problem-definition” (M) and to “look at possible interventions” (M). ‘Treatable suffering(M)’ should be addressed before considering euthanasia. However, this opinion comes with an ethical *side-note* of “proportionality“ (E)*:* “motivated deviation from the guideline problem behaviour is good medical practice” (E).

It is argued that in case of ‘refractory problem behaviour’ additional use of the ‘guide on palliative sedation’ should also be part of ”good care” (L). However, palliative sedation should aim at “reducing suffering and not initiating death” (M)*;* in other words: “Palliative sedation must not become disguised euthanasia” (E).

## Discussion

This article sought consensus from medical, ethical and legal perspectives on handling ethical dilemmas in euthanasia requests for dementia cases based on advance directives. It highlights challenges faced by physicians in meeting the due care criteria when replacing an oral request with an AED. Communication is highly important, with consensus on acknowledging current expressions of individuals with dementia and prioritising a wish to live over an AED. AEDs can support patients expressions in the decision-making process, but AEDs linking euthanasia to nursing home admittance are considered infeasible. Substitute decision-making in requests for euthanasia is ruled out, while the delicate balance between stringent requirements in cases of euthanasia and respecting the human rights of people with disabilities in the context of decisional capacity is highlighted. Despite varying expertise in problem behaviour guidelines, there is a consensus on their relevance in addressing ‘treatable suffering’ caused by problem behaviour before considering euthanasia.

Our research shows that euthanasia in dementia based on an AED (art. 2.2 Euthanasia law) presents primarily challenges for physicians in daily practice. This finding can be substantiated by existing discrepancies between the ‘legal possibility’ of euthanasia based on an AED and the ‘ethical justification’ to fulfil the due care criteria. While the Euthanasia law sets open norms, physicians are responsible to fulfil the criteria and substantiate their choices.^7^ Put differently: while ‘mutatis mutandis’ suggests possible applicability^3^ of the due care criteria in AED-based euthanasia, physicians express the practical complexity, some even deeming it ‘impossible’. This complexity may contribute to the low numbers of AED-based euthanasia.^10 33^ Substantiation of the found complexities in our study press for understanding and support for the cautiousness of physicians facing such dilemmas in practice.

In line with previous research,^19^ our study shows that actual (non-verbal) patient–physician communication is for most of our panellists a precondition to consider AED-based euthanasia. Both physicians and ethicists stress the significance of interpreting and valuing the expressions and behaviours of individuals with dementia, prioritising the wish to live over an AED. To consider euthanasia, most medical panellists indicate they need some form of (behavioural) expressions, of the wish for euthanasia and the experience of unbearable suffering. Moreover, the fourth due care criterion of the Euthanasia law stipulates that patient and physician must jointly conclude that reasonable alternatives are lacking. Resonance of all of these aspects within the expressions of a person with dementia is according to both medical and ethical panellist relevant given the possibility of shifting boundaries. Another line of reasoning, substantiated by our legal panellists, is that labelling patient–physician communication as a necessity would undermine the essence of article 2.2 of the Euthanasia law, which states that an oral request can be replaced by an AED in case the patient is ‘no longer able to express his will’*.*^1^ But what exactly is meant by this wording? This raises the complex issue of decisional incapacity in euthanasia decision-making.

Although no consensus was reached on the topic of decisional (in)capacity with our study’s statements, panellists emphasised its need for careful consideration. Whereas the Euthanasia law may suggest differently, decisional incapacity is not abrupt, rather a gradual, even fluctuating process of diminishing decision-making abilities.^34 35^ Moreover, decisional capacity is a task-specific and risk-dependent concept with a variable threshold: as the consequences of a decision become more serious, higher demands are placed on decisional capacity.^34^ In practice, this corresponds to a high threshold for euthanasia decisions, contrasting with a low threshold for decisional capacity regarding a wish to live. This preference for life over an AED is supported by all but our legal panellists. Furthermore, panellists, citing human rights, caution against easily labelling individuals as decisional incapacitated, which can be attributed to the United Nations Convention on the Rights of Persons with Disabilities (UNCPRD).^36^ This convention advocates for a shift from substitute decision-making towards supportive decision-making, emphasising actual wishes and preferences of people with decision-making disabilities. Within this context, all panellist groups agree that substitute decision-making is infeasible for highly personal decisions like euthanasia, which resonates with the decisional capacity guideline.^34^

This does not mean dementia patients’ representatives with an AED play no role at all in euthanasia decisions. As articulated by the KNMG and quoted by both a medical and legal panellist: “The doctor can take the opinions of the relatives into consideration in the deliberations, but these can never replace the requirement of a voluntary and well-considered request from the patient*”*.^20^ Likewise, while communication becomes challenging, the AED serves as a crucial source of information. In this context, panellists note that broad consultation helps assess the alignment of behaviour and expressions of the person with dementia with their earlier AED, but they also refer to it as a ‘slippery slope’. Ethical dilemmas may arise in case of doubts about this alignment—for example, less obvious signs of suffering or differences in interpretation of the patient’s ‘voice’. Under such circumstances, physicians may tend towards ‘in dubio abstine’ regarding euthanasia, a highly valued medical ethical principle guiding practice under circumstances of uncertainty.^37^

Our study’s strength lies in bringing together medical, ethical and legal expertise, all contributing to the debate. Through carefully sampling our panellists, we believe we have effectively minimalised the potential pitfalls associated with biased expert panels, a critical aspect in Delphi studies. The study is limited by not reaching consensus on all statements, and not having included the perspective of people with dementia themselves. Both these limitations may ask for further exploration in future research. Although our research was conducted within the framework of Dutch Euthanasia Law and the consensus statements may not be directly applicable to other national or cultural contexts, the findings could still contribute to broader discussions on euthanasia in dementia, particularly in countries with existing legislation or those considering legislation on euthanasia and AEDs.

## Conclusion

This study revealed differences in opinions and argumentations regarding ethical dilemmas in AED-based euthanasia in dementia. Legal experts tend to lean towards a theoretical interpretation of the open standards set by the Euthanasia law, while physicians, often supported by ethicists, shed light on the practical dilemmas it poses. Despite these differences, consensus was reached on multiple topics regarding AED-based euthanasia. This consensus may assist physicians in establishing their personal boundaries in AED-based euthanasia cases. Understanding the arguments related to encountered dilemmas and the fulfilment of due care criteria can both justify decisions in euthanasia cases based on an AED and support decisions to refrain from euthanasia.

## Supplementary material

10.1136/jme-2024-110276 online supplemental appendix 1

## Data Availability

No data are available. All data relevant to the study are included in the article or uploaded as supplementary information.

## References

[1] (2002). The termination of life on request and assisted suicide (review procedures) act. https://wfrtds.org/dutch-law-on-termination-of-life-on-request-and-assisted-suicide-complete-text/.

[2] Regional Euthanasia Review Committees (2024). https://english.euthanasiecommissie.nl/.

[3] Policy and regulation Aanwijzing 3.32 terminologie “van (overeenkomstige) toepassing”. https://www.kcbr.nl/beleid-en-regelgeving-ontwikkelen/aanwijzingen-voor-de-regelgeving/hoofdstuk-3-aspecten-van-vormgeving/ss-33-aanhaling-en-verwijzing/aanwijzing-332-terminologie-van-overeenkomstige-toepassing.

[4] Coers DO, de Boer ME, Sizoo EM (2023). Dealing with requests for euthanasia in incompetent patients with dementia. Qualitative research revealing underexposed aspects of the societal debate. Age Ageing.

[5] Menzel PT, Steinbock B (2013). Advance directives, dementia, and physician-assisted death. J Law Med Ethics.

[6] van Delden JJM (2004). The unfeasibility of requests for euthanasia in advance directives. J Med Ethics.

[7] Coers DO, Scholten SH, de Boer ME (2024). A qualitative focus group study on legal experts’ views regarding euthanasia requests based on an advance euthanasia directive. BMC Med Ethics.

[8] de Boer ME, Dröes R-M, Jonker C (2010). Advance directives for euthanasia in dementia: do law-based opportunities lead to more euthanasia?. Health Policy.

[9] Verenso (2018). Euthanasie bij gevorderde dementie.

[10] RERC (2022). Annual report.

[11] Gastmans C, De Lepeleire J (2010). Living to the bitter end? A personalist approach to euthanasia in persons with severe dementia. Bioethics.

[12] Groenewoud AS, Leijten E, van den Oever S (2022). The ethics of euthanasia in dementia: A qualitative content analysis of case summaries (2012-2020). J Am Geriatr Soc.

[13] Hertogh CMPM, de Boer ME, Dröes R-M (2007). Would we rather lose our life than lose our self? Lessons from the Dutch debate on euthanasia for patients with dementia. Am J Bioeth.

[14] Coers DO, Scholten SH, de Boer ME (2024). Euthanasia requests based on an advance euthanasia directive. A qualitative focus group study on the views of legal experts [Submitted].

[15] de Boer ME, Hertogh CMPM, Dröes R-M (2010). Advance directives in dementia: issues of validity and effectiveness. Int Psychogeriatr.

[16] Dresser R, Whitehouse PJ (1994). The incompetent patient on the slippery slope. Hastings Cent Rep.

[17] Dworkin R (1986). Autonomy and the demented self. Milbank Q.

[18] de Beaufort ID, van de Vathorst S (2016). Dementia and assisted suicide and euthanasia. J Neurol.

[19] de Boer ME, Dröes R-M, Jonker C (2011). Advance directives for euthanasia in dementia: how do they affect resident care in Dutch nursing homes? Experiences of physicians and relatives. J Am Geriatr Soc.

[20] KNMG KNMG-standpunt beslissingen rond het levenseinde. Contract no.: May.

[21] RERC Judgements. https://www.euthanasiecommissie.nl/uitspraken-en-uitleg.

[22] (2020). Criminal Case, ECLI:NL:HR:2020:712.

[23] (2020). Disciplinary case, ECLI:NL: HR:2020:713.

[24] Hsu C-C, Sandford BA (2007). The Delphi Technique: Making Sense of Consensus. Pract Assess Res Eval.

[25] Kerlinger FN (1973). Foundation of behavioural research.

[26] Nasa P, Jain R, Juneja D (2021). Delphi methodology in healthcare research: How to decide its appropriateness. World J Methodol.

[27] Barrett D, Heale R (2020). What are Delphi studies?. Evid Based Nurs.

[28] Taylor E (2020). We Agree, Don’t We? The Delphi Method for Health Environments Research. HERD.

[29] Castor EDC [computer software]. Ciwit B.V.

[30] Coers DO, Sizoo EM, Bloemen M (2024). Navigating Dilemmas on Advance Euthanasia Directives of Patients with Advanced Dementia. J Am Med Dir Assoc.

[31] Verenso (2020). Palliatieve sedatie bij refractair probleemgedrag bij mensen met dementie.

[32] Software V (2021). MAXQDA 2022 [computer software].

[33] RERC (2021). Annual report.

[34] SKILZ (2024). Handreiking Beslisvaardigheid en wilsbekwaamheid.

[35] van der Steen JT, Nakanishi M, Van den Block L (2024). Consensus definition of advance care planning in dementia: A 33-country Delphi study. Alzheimers Dement.

[36] Nations U (2006). Convention on the rights of persons with disabilities (CRPD).

[37] Griffith J, Bood A, Weyers H (1998). Euthanasia and Law in the Netherlands.

