# Supplementary material for: Seeking consensus on dilemmas related to euthanasia in dementia based on an advance directive: a Delphi study from a medical, ethical and legal perspective
Source: J Med Ethics. 2025 Jan 28;51(9):e110276. doi: 10.1136/jme-2024-110276 (PMC12418533; doi:10.1136/jme-2024-110276)
Supplement: online supplemental appendix 1 [file jme-51-9-s001.pdf]

Appendix

Characteristics participating panellists (n=29)

Expertise group	*Age range*	*Gender**	*Role (relevant for Delphi study)*	*Geographical region*
Legal	*60-69*	*M*	*Associate Professor Criminal Law*	*West Netherlands*
Legal	*60-69*	*M*	*Professor of Health Law*	*West Netherlands*
Legal	*70-79*	*M*	*Endowed professor of Legal Protection for the Elderly and Adults with Disabilities*	*West Netherlands*
Legal	*30-39*	*F*	*Lecturer Health Criminal Law*	*West Netherlands*
Legal	*60-69*	*M*	*Professor of Health Law*	*Flanders, Belgium*
Legal	*50-59*	*F*	*Professor of Law and Health*	*West Netherlands*
Legal	*50-59*	*M*	*Assistant Professor of Health law*	*North Netherlands*
Legal	*55-60*	*F*	*Health Lawyer*	*West Netherlands*
Legal	*60-69*	*F*	*Professor of Forensic Medicine, Health and Criminal Justice*	*South Netherlands*
Legal	*70-79*	*F*	*Lecturer Department of Law*	*North Netherlands*
Ethical	*60-69*	*M*	*Professor of Healthcare Ethics*	*West Netherlands*
Ethical	*80-89*	*M*	*Emeritus Professor of Ethics*	*West Netherlands*
Ethical	*50-59*	*F*	*Professsor of Medical Philosophy and Ethics*	*West Netherlands*
Ethical	*50-59*	*M*	*Professor of Medical Ethics*	*Flanders, Belgium*
Ethical	*50-59*	*F*	*Associate professor of Medical Ethics*	*West Netherlands*
Ethical	*60-69*	*F*	*Associate professor of Medical Ethics*	*North Netherlands*
Ethical	*50-59*	*F*	*Professor of Healthcare Ethics*	*East Netherlands*
Ethical	*30-39*	*F*	*Palliative care researcher*	*Flanders, Belgium*
Ethical	*50-59*	*F*	*Associate professor Medical Ethics*	*West Netherlands*
Ethical	*40-49*	*F*	*Assistant Professor, Ethics, Law & Medical Humanities*	*West Netherlands*
Medical	*50-59*	*F*	*Lecturer Elderly Care; Elderly Care Physician*	*East Netherlands*
Medical	*50-59*	*F*	*Elderly Care Physician*	*West Netherlands*
Medical	*40-49*	*F*	*Professor of Medicine for older people; Elderly Care Physician*	*South Netherlands*

Medical	*30-39*	*F*	*Elderly Care Physician; senior researcher*	*East Netherlands*
Medical	*60-69*	*F*	*Elderly Care Physician*	*East Netherlands*
Medical	*30-39*	*F*	*Elderly Care Physician*	*West Netherlands*
Medical	*50-59*	*F*	*Elderly Care Physician*	*East Netherlands*
Medical	*50-59*	*F*	*Lecturer Elderly Care Medicine; Elderly Care Physician*	*South Netherlands*
Medical	*50-59*	*F*	*Elderly Care Physician*	*North Netherlands*

** M=Male; F=Female*

*Number of panelists over three Delphi rounds**

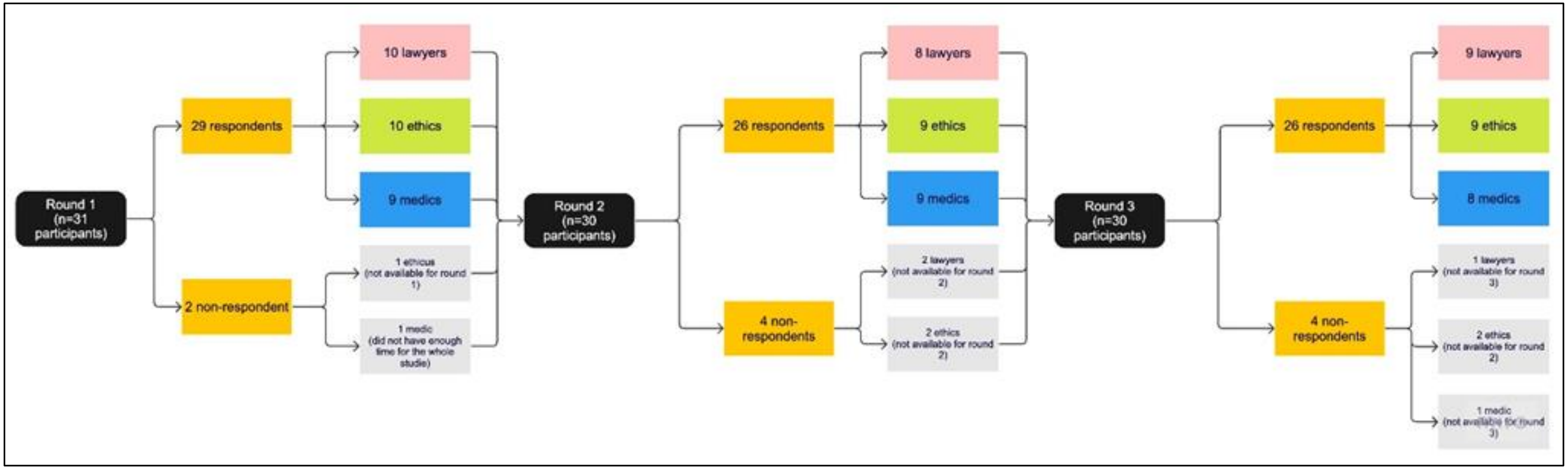

* One panelist dropped out from all three Delphi rounds, and was left out in all equations

*Summary statements and consensus per Delphi round**

Number	Round	Statement	Consensus
Statement 1	Round 1	The wording that, in the case of the application of Article 2.2 of the Euthanasia Law, the due care criteria apply 'mutatis mutandis', provides insufficient clarity	71%
	Round 2	''	63%
	Round 3	The wording that, in the case of the application of Article 2.2 of the Euthanasia Law, the due care criteria apply 'mutatis mutandis', does not provide sufficient clarity for physicians in practice	73%
Statement 2	Round 1	The current expressions and behaviors of the (decisional incapacitated) patient should never be ignored	80%
	Round 2	''	87%
Statement 3	Round 1	Bij een wens tot leven (afgeleid uit actuele uitingen of gedragingen – hoe ongearticuleerd ook) moet de patiënt laagdrempelig als wilsbekwaam worden beschouwd, en moet de wens tot leven prevaleren boven een euthanasieverklaring	61%
	Round 2	Een wens tot leven (afgeleid uit actuele uitingen of gedragingen – hoe ongearticuleerd ook) moet prevaleren boven een schriftelijke euthanasieverklaring	82%
Statement 4	Round 1	Without (non-verbal) communication and mutual understanding between physician and patient proceeding to fulfillment of the advance euthanasia directive is not possible	76%
	Round 2	Without (non-verbal) communication and mutual understanding between physician and patient proceeding proceeding to performance of euthanasia is not possible	56%
	Round 3	Current (non-verbal) communication between doctor and patient is a prerequisite for proceeding with euthanasia	59%
Statement 5	Round 1	If mutual (non-verbal) communication is not possible, the physician can obtain clarity about the wish of the patient through consensual assessment *[the essence of consensual assessment is aimed at obtaining consensus between the actual expressions of the patient and his/her prior advance directive]*	54%
	Round 2	If mutual (non-verbal) communication is not possible, the physician can obtain clarity through a broad orientation with consultation from multiple sources (e.g., family, caregivers, other healthcare professionals) regarding whether there is agreement between the patient's current expressions and their earlier advance directive	69%
	Round 3	If mutual (non-verbal) communication is not possible, the physician can obtain clarity through a broad orientation with consultation from multiple sources (e.g., family, caregivers, other healthcare professionals) regarding whether there is agreement between the patient's current expressions and behaviors and their earlier advance directive	64%
Statement 6	Round 1	If a patient with dementia and a written advance euthanasia directive expresses an oral request, the threshold for decisional capacity should be high, whereby the patient should at least be able to confirm the advance euthanasia directive.	33%
Statement 6a	Round 2	If a patient with dementia and a written advance euthanasia directive expresses an oral request, the same requirements for decisional capacity apply for this request as for an oral request of a patient without such an advance euthanasia directive.	49% (51% disagree)
	Round 3	In general, strict requirements for decisional capacity apply to oral requests for euthanasia, where in the case of dementia, it makes no difference whether the patient in question has a written euthanasia directive or not.&	25% (76% disagree)
Statement 6b	Round 2	If a patient with dementia makes an oral request for euthanasia, a written advance euthanasia directive can be supportive in the discussion and decision-making regarding that request.	92%
Statement 7	Round 1	Advance directives with formulations such as 'if I have to be admitted to a nursing home, then I want euthanasia' are infeasible	93%
	Round 2	"	92%
Statement 8	Round 1	Substitute decision-making is not an option for highly personal decisions such as euthanasia or assisted suicide	90%

		[in substitute decision-making, the decision is taken over by the legal representative]	
	Round 2	“	88%
Statement 9	Round 1	If suffering arises from problem behavior, the professional guideline problem behavior should be followed before considering euthanasia.	78%
	Round 2	If suffering arises from problem behaviour, application of the professional guideline problem behaviour is essential to be able to assess whether or not there is hopelessness of suffering.	83%
	Round 3	If suffering arises from problem behaviour, application of the professional guideline problem behaviour is strongly recommended to be able to assess whether or not there is hopelessness of suffering	96%
Statement 10	Round 1	In case of refractory problem behaviour, the possibilities of the the guide on palliative sedation in case of refractory problem behaviour should also be explored.	76%
	Round 2	In case of refractory problem behaviour (i.e., problem behaviour that cannot be alleviated by optimal care and treatment), it is the professional standard to also consider and discuss the options outlined in the guide on palliative sedation in case of refractory problem behaviour	82%
	Round 3	”	84%

* Statements highlighted in green reached consensus; statements highlighted in orange did not reach consensus
